# Comparison of the information provided by electronic health records data and a population health survey to estimate prevalence of selected health conditions and multimorbidity

**DOI:** 10.1186/1471-2458-13-251

**Published:** 2013-03-21

**Authors:** Concepción Violán, Quintí Foguet-Boreu, Eduardo Hermosilla-Pérez, Jose M Valderas, Bonaventura Bolíbar, Mireia Fàbregas-Escurriola, Pilar Brugulat-Guiteras, Miguel Ángel Muñoz-Pérez

**Affiliations:** 1 Institut Universitari d’Investigació en Atenció Primària Jordi Gol (IDIAP Jordi Gol), Barcelona, Spain; 2 Universitat Autònoma de Barcelona, Bellaterra, Spain; 3 Institut Català de la Salut, Catalunya, Spain; 4 Hospital de Campdevànol, Campdevànol, Spain; 5 NIHR School for Primary Care Research, Health Services and Policy Research Group, Department of Primary Care Health Sciences, University of Oxford, Oxford, UK; 6 Departament de Salut, Generalitat de Catalunya, Barcelona, Spain

**Keywords:** Multimorbidity, Electronic health records, Health surveys, Primary health care

## Abstract

**Background:**

Health surveys (HS) are a well-established methodology for measuring the health status of a population. The relative merit of using information based on HS versus electronic health records (EHR) to measure multimorbidity has not been established. Our study had two objectives: 1) to measure and compare the prevalence and distribution of multimorbidity in HS and EHR data, and 2) to test specific hypotheses about potential differences between HS and EHR reporting of diseases with a symptoms-based diagnosis and those requiring diagnostic testing.

**Methods:**

Cross-sectional study using data from a periodic HS conducted by the Catalan government and from EHR covering 80% of the Catalan population aged 15 years and older. We determined the prevalence of 27 selected health conditions in both data sources, calculated the prevalence and distribution of multimorbidity (defined as the presence of ≥2 of the selected conditions), and determined multimorbidity patterns. We tested two hypotheses: a) health conditions requiring diagnostic tests for their diagnosis and management would be more prevalent in the EHR; and b) symptoms-based health problems would be more prevalent in the HS data.

**Results:**

We analysed 15,926 HS interviews and 1,597,258 EHRs. The profile of the EHR sample was 52% women, average age 47 years (standard deviation: 18.8), and 68% having at least one of the selected health conditions, the 3 most prevalent being hypertension (20%), depression or anxiety (16%) and mental disorders (15%). Multimorbidity was higher in HS than in EHR data (60% vs. 43%, respectively, for ages 15-75+, P <0.001, and 91% vs. 83% in participants aged ≥65 years, P <0.001). The most prevalent multimorbidity cluster was cardiovascular. Circulation disorders (other than varicose veins), chronic allergies, neck pain, haemorrhoids, migraine or frequent headaches and chronic constipation were more prevalent in the HS. Most symptomatic conditions (71%) had a higher prevalence in the HS, while less than a third of conditions requiring diagnostic tests were more prevalent in EHR.

**Conclusions:**

Prevalence of multimorbidity varies depending on age and the source of information. The prevalence of self-reported multimorbidity was significantly higher in HS data among younger patients; prevalence was similar in both data sources for elderly patients. Self-report appears to be more sensitive to identifying symptoms-based conditions. A comprehensive approach to the study of multimorbidity should take into account the patient perspective.

## Background

Multimorbidity is “the co-occurrence of multiple medical conditions within one person without any reference to an index condition” [1]. Multimorbidity is very common among people using primary health care services and has a serious impact on the utilization of health resources [2,3]. Although there is emerging evidence for the prevalence of multimorbidity based on medical records data, there is fundamental lack of knowledge about its prevalence based on patient self-report [4]. Many long-term surveys have been designed to determine the impact, needs and magnitude of health problems and the role of health programs and health care providers in addressing these problems [5]. Since 1994, the Government of Catalonia (North-West Spain) has periodically measured the health of a representative sample of the population with the Health Survey for Catalonia [6]. Although such self-reports are reasonably accurate to estimate the prevalence of certain health conditions and for routine screening exams, some variability exists when they are compared to the information registered in medical records [7-10].

In general, consensus methods to define multimorbidity prevalence do not exist. In two recent reviews the prevalence of chronic health conditions was higher in medical records than in other data sources, such as administrative data or health surveys (HS) [11,12]. Other studies report that most of the more symptomatic chronic diseases are more poorly recorded in electronic health records (EHR) [13].

This discrepancy has not been fully addressed in the literature by studies that compare the prevalence of multimorbidity in EHR and in patient surveys. Therefore, we designed a study with two objectives: 1) to measure and compare the prevalence and distribution of multimorbidity in the population and in patients seen in primary health care, and 2) to test two specific hypotheses about potential differences between HS and EHR reporting of diseases with a symptoms-based diagnosis and those requiring diagnostic testing.

## Methods

### Study design

Cross-sectional study of residents of Catalonia, a region of northeast Spain with a population of 7,475,420 persons according to the 2009 population census.

### Data sources

Self-reported chronic morbidity was obtained from the Health Survey for Catalonia database (2006). In the survey, respondents reported whether or not they had each of 27 selected health problems (see below) [6]. The HS was administered to a representative sample of the Catalan population identified through multistage sampling and stratified by age group, sex and municipal stratum of the Territorial Health Authority (*Gobierno Territorial de Salud*). Calculation of the confidence intervals (CIs) took into account the sampling design effects. The sample of 18,126 individuals included 15,926 individuals aged 15 years or older and 2,200 children younger than 15 years [14]. Only the first age group was included in this study.

The selection process was based on the 27 health problems included in the Health Survey (HS) interview, as follows: The interviewer asks if the individual has any chronic health problem, and then reads the list of 27 health problems, each of which has a unique code.

Registered morbidity was collected for each individual from the primary care EHR system administered by the Catalan Institute of Health. The primary care structure in the region comprises 358 primary care practices (PCP) composed of health professionals and support staff who are responsible for the health care of the population in their assigned geographic area. The Catalan Institute of Health manages 274 PCP (76.5%); the remaining centres are managed by other health care entities. Each PCP has at least three (and an average of 12) basic care units, defined as one general practitioner (GP) and one nurse providing care for an assigned set of patients. The Information System for the Development of Research in Primary Care (SIDIAP) database comprises the anonymized clinical information coded in the corresponding EHR of all 274 PCPs. Their 3,414 basic care units are assigned an adult population of 4,859,725 persons. A SIDIAP sample of 40% of the basic care units meeting the highest quality criteria was selected (SIDIAP Q), yielding a total of 1,936,443 patients. Therefore, SIDIAP Q contains clinical data from EHR for those patients attended by the 1,365 GPs in Catalonia who achieve the highest quality of clinical data recording in their EHR. This methodology diminishes potential selection bias and facilitates accurate estimation of prevalence rates and other results [15,16]. The sample is representative of the general Catalan population in terms of geography, age and sex distributions, as recorded in the official 2009 census [17]. We selected patients aged 15 years or older who were alive and permanently registered in their PCP on 31 December 2009, for a study population of 1,597,258.

### Health conditions and multimorbidity

This study focused on 27 chronic health problems for which there was HS information. Patient diagnoses in the EHR data are recorded using International Statistical Classification of Diseases and Related Health Problems, 10th Revision (ICD-10) codes [18]. A mapping process was designed to permit comparison of entries in the two data sources. Four experienced GPs and one public health specialist assigned all of the ICD-10 codes for diagnoses corresponding to the 27 health conditions obtained from HS data (see Additional file 1: Appendix 1 for details on the Health Survey for Catalonia). Disagreements were resolved by consensus.

Multimorbidity was defined as the presence of two or more of the 27 targeted health conditions in one individual. Prevalent combinations of these conditions constitute patterns of multimorbidity [19] that were further analysed.

In designing this study, we hypothesized that over- and underreporting of any condition in each data source may be associated with the information used for diagnosis and management, i.e., mainly based on symptoms or on diagnostic test results. Therefore, we classified these chronic conditions in two groups based on the diagnostic approach. Group 1 (13 conditions predominantly based on diagnostic tests) includes anaemia, asthma, cardiac disease, cerebrovascular diseases, chronic obstructive pulmonary disease, diabetes mellitus, hypercholesterolemia, hypertension, myocardial infarction, malignant tumours, osteoporosis, peptide ulcer and thyroidal diseases. Group 2 (14 conditions predominantly based on symptoms) includes back pain; cataracts; chronic allergies; chronic constipation; depression or anxiety; haemorrhoids; mental disorders; migraine or frequent headaches; neck pain; osteoarthritis, arthritis or rheumatism; circulation disorders (other than varicose veins); prostatic disorders; skin diseases and varicose veins.

### Confidentiality and ethical issues

The study protocol was approved by the Committee on the Ethics of Clinical Research of the Jordi Gol i Gurina Foundation of the University Institute for Research in Primary Care (*Institut Universitari d’Investigació en Atenció Primària (IDIAP) Jordi Gol*)*.* All data were anonymized and the confidentiality of medical records was respected at all times in accordance to Spanish law [20].

### Statistical methods

The crude prevalence of multimorbidity was calculated overall and stratified by age group and sex. The presence of each of the selected health conditions was considered as a binary variable. We provide a descriptive analysis, including 95% CIs from each source, as calculated separately and under the assumption of a binomial distribution.

We calculated the number of selected health conditions in every patient, and then determined which of the conditions contributed to multimorbidity in each database (HS and EHR). We further explored whether differences existed between the two information sources, calculating ratios between crude prevalences in the HS and EHR.

We then calculated the frequencies in EHR data of all potential multimorbidity patterns, defined as the combination of 2 or 3 of the 27 health problems assessed in the study. Calculations were based on the following formula: C n,r = n!/r!(n-r)! (where C is the number of combinations, n = number of elements to combine (27 health problems), and r = the size of the subgroups of elements (i.e., 2 or 3 items in our case). There are 351 possible combinations of 2 conditions and 2,925 combinations of 3 conditions.

We tested two complementary hypotheses: a) Selected health conditions requiring diagnostic tests were more prevalent in the EHR than in the HS data, and b) Symptoms-based health problems were more prevalent in the HS data than in the EHR.

Statistical significance was set at α = 0.005 and analysis was performed using the Survey Analysis Package of Stata Statistical Software (Stata), release 10.

## Results

### Measuring prevalence of multimorbidity in health survey data

Of the 15,926 interviews, 50.5% were women and the age distribution was 49.6% aged 15–44, 28.0% aged 45–64, and 22.4% 65 years or older (similar to the Catalan census distribution). At least 77.4% of the general population sample reported at least one of the morbidities listed on the HS, with higher prevalence in women (83.0% vs. 71.6% in men, P < 0.001), rising to 97.5% in patients aged 65 years or older.

Women most frequently reported back pain (29.6%), neck pain (27.4%); osteoarthritis, arthritis or rheumatism (22.7%); circulation disorders (other than varicose veins) (20.0%); hypertension (19.7%); varicose veins (19.3%) and migraine or frequent headaches (18.9%) (Table 1).

**Table 1:** **Morbidity in health survey and electronic health records and calculation of 95**% **confidence interval**

	**Health survey (HS)**		**Electronic health records (EHR)**		Ratio HS/EHR*
	**%**	**CI†95%**	**%**	**CI†95%**	
**Anaemia**	7.3	[6.9 - 7.7]	4.6	[4.6- 4.6]	**1.6**
**Asthma**	6.1	[5.7- 6.5]	4.1	[4.1- 4.1]	**1.5**
**Back pain**	29.6	[28.9- 30.3]	13.6	[13.5- 13.6]	**2.2**
**Cardiac disease**‡	6.5	[6.1- 6.9]	6.6	[6.5- .6.6]	**0.9**
**Cataracts**	8.2	[7.8- 8.6]	3.2	[3.2- 3.3]	**2.5**
**Cerebrovascular disease**	1.8	[1.6- 2.0]	1.3	[1.3- 1.3]	**1.4**
**Chronic allergies**	16.2	[15.6- 16.8]	3.2	[3.1- 3.2]	**5.1**
**Chronic constipation**	9.4	[9.0- 9.9]	2.7	[2.7- 2.7]	**3.5**
**COPD**§	6.2	[5.8- 6.6]	3.8	[3.7- 3.8]	**1.6**
**Depression or anxiety**	17.5	[16.9- 18.1]	15.9	[15.8- 15.9]	**1.1**
**Diabetes mellitus**	5.9	[5.5- 6.3]	7.6	[7.6- 7.7]	**0.8**
**Haemorrhoids**	12.7	[12.2- 13.2]	3.3	[3.3- 3.4]	**3.8**
**Hypercholesterolemia**	14.9	[14.4- 15.5]	9.8	[9.7- 9.8]	**1.5**
**Hypertension**	19.7	[19.1- 20.3]	20.4	[20.4- 20.5]	**1.0**
**Mental disorders**||	2.6	[2.4- 2.9]	14.8	[14.8-14.9]	**0.2**
**Migraine or frequent headaches**	18.9	[18.3- 19.5]	5.2	[5.1- 5.2]	**3.6**
**Myocardial infarction**	2.2	[2.0- 2.4]	2.2	[2.1- 2.2]	**1.0**
**Neck pain**	27.4	[26.7- 28.1]	5.7	[5.7- 5.8]	**4.8**
**Malignant tumours**	3.0	[2.7- 3.3]	3.6	[3.6- 3.6]	**0.8**
**Osteoarthritis, arthritis or rheumatism**	22.7	[22.1- 23.4]	11.3	[11.2- 11.3]	**2.0**
**Osteoporosis**	5.6	[5.2- 6.0]	3.9	[3.8- 3.9]	**1.4**
**Circulation disorders** ¶	20.0	[19.4- 20.6]	1.1	[1.0-1.1]	**18.2**
**Peptic ulcers**	5.8	[5.4- 6.2]	1.7	[1.7- 1.7]	**3.4**
**Prostatic disorders**	8.4	[7.8- 9.0]	9.1	[9.0- 9.1]	**0.9**
**Skin diseases**	7.5	[7.1- 7.9]	9.7	[9.7- 9.8]	**0.8**
**Thyroidal diseases**	4.4	[4.1- 4.7]	2.6	[2.6- 2.6]	**1.7**
**Varicose veins**	19.3	[18.7- 19.9]	5.6	[5.5- 5.6]	**3.5**

*Ratio of HS and EHR prevalence. † CI: confidence interval. ‡ Except myocardial infarction. §Chronic obstructive pulmonary disease. || Except depression and anxiety. ¶ Excluding varicose veins.

### Measuring prevalence of morbidities in electronic health records

Of the 1,597,258 records included, 52.4% were women and the age distribution was 50.9% aged 15–44, 28.8% aged 45–64, and 20.2% 65 years or older, similar to that obtained in the HS and in the Catalan census.

At least 67.7% of the records included at least one of the selected health conditions. In patients aged 65 and older, this percentage increased to 94.1%. The most frequently recorded health problem was hypertension (20.4%), followed by depression or anxiety (15.9%), mental disorders (14.8%) and back pain (13.6%) (Additional file 2: Appendix 2).

By age group, the most prevalent diseases were mental and skin diseases in the youngest group and hypertension in those aged 45–64 years (approximately 25% prevalence); in the oldest group, more than half have hypertension and more than a quarter have osteoarthritis, arthritis or rheumatism (see Additional file 2: Appendix 2 for more detail).

Anaemia, depression or anxiety, migraine or frequent headaches, osteoporosis, thyroidal diseases and varicose veins were more than twice as prevalent in women, whereas COPD and peptic ulcer were more frequent in men.

### Comparison of prevalence of multimorbidity

**Figure 1:**
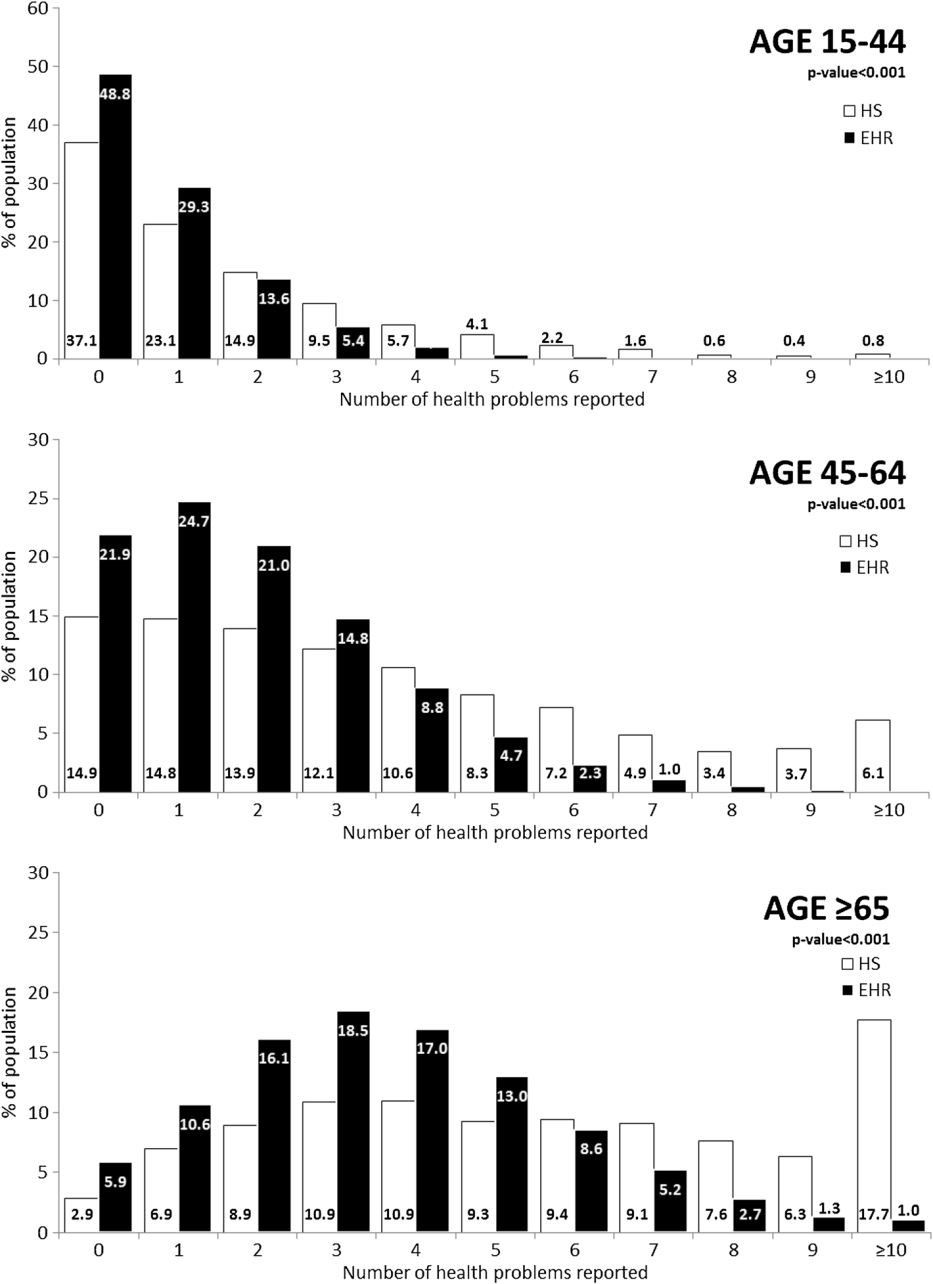
Number of health problems, distributed by source (HS and EHR) in strata of increasing age.

The median number of health problems registered in EHR was 1 (Interquartile Range: 0–3); 2 and 3 health problems were registered for 16.3% and 10.8% of the population, respectively. Figure 1 shows the differences in the number of health problems, stratified by age group and by information source (HS or EHR). In both sources, older people had a higher number of chronic conditions.

Comparison of multimorbidity prevalence obtained from the two sources is described in Table 2. In all four age groups, the prevalence was higher in the self-reported HS data; notably, however, this difference between HS and EHR data decreases in older age groups (Table 2).

**Table 2:** Prevalence of multimorbidity in health survey and electronic health records

	**Healthsurvey(N = 15,926)**		**Electronichealth records (N = 1,597,258)**		
**Age group**	**N**	**Multimorbidity prevalence (%, 95% CI)**	**N**	**Multimorbidity prevalence (%, 95% CI)**	**Prevalence difference (%, 95% CI)**
**15-44**	7,894	39.7 (38.4-41.0)	813,437	21.6 (21.5-21.7)	18.1 (16.8-19.4)
**45-64**	4,466	70.3 (69.3-71.1)	460,496	52.9 (52.8-53.1)	17.4 (16.5-18.3)
**65-74**	1,703	87.5 (86.7-87.9)	162,591	79.8 (79.6-80.0)	7.7 (7.1-8.3)
**≥75**	1,863	92.9 (91.7-92.5)	160,734	87.2 (87.0-87.3)	5.7 (5.3-6.1)

### Multimorbidity patterns in EHR data

Of the 351 possible combinations of two conditions and 2,925 possibilities for three conditions, we only provide the most prevalent results. Table 3 lists by sex and age group the most common pairs and triads of possible combinations of the 27 health problems surveyed in EHR data.

**Table 3:** Clusters of two and three health problems by age group and sex in electronic health records

**Age group**	**Male**	**Female**
**15-44**	**Two:** Depression or anxiety & mental disorder (2.5%)	**Two:** Depression or anxiety & mental disorder (3.8%)
	**Three:** Depression or anxiety & mental disorder & back pain (0.5%)	**Three:** Depression or anxiety & mental disorder & back pain (0.8%)
**45-64**	**Two:** Hypertension & diabetes mellitus (6.2%)	**Two:** Depression or anxiety & mental disorder (9.9%)
	**Three:** Hypertension & diabetes mellitus & hypercholesterolemia (1.5%)	**Three:** Hypertension & depression or anxiety mental disorder & osteoarthritis, arthritis or rheumatism (2.0%)
**65-74**	**Two:** Hypertension & prostatic disorder (17.5%)	**Two:** Hypertension & osteoarthritis, arthritis or rheumatism (22.4%)
	**Three:** Hypertension & prostatic disorder& osteoarthritis, arthritis or rheumatism (5.5%)	**Three:** Hypertension & osteoarthritis, arthritis or rheumatism & depression or anxiety (7.1%)
**≥75**	**Two:** Hypertension & prostatic disorders (28.2%)	**Two:** Hypertension & osteoarthritis, arthritis or rheumatism (32.9%)
	**Three:** Hypertension & prostatic disorder & cardiac disease (10.9%)	**Three:** Hypertension & osteoarthritis, arthritis or rheumatism & cardiac disease (11.1%)

### Comparison of perceived and recorded data

Some health problems were more prevalent in the HS than in EHR data. For 80% of all health problems, the self-reported morbidity substantially exceeded the EHR data. Table 1 shows these results and the corresponding 95% CIs for each source. Differences were especially high for circulation disorders (other than varicose veins), chronic allergies, neck pain, haemorrhoids, migraine or frequent headaches and chronic constipation. On the other hand, EHR data showed a higher prevalence of mental disorders, diabetes mellitus, malignant tumours and skin diseases.

The first hypothesis tested (i.e., conditions based on test results will be more prevalent in EHR) was confirmed only in four of the 13 test-based conditions (30.7%, CI 95%: 9.1%-61.9%), whereas the second hypothesis (symptomatic conditions would be more reflected in HS) was confirmed in 10 of the 14 symptomatic conditions (71.4%, CI 95%: 41.9%-91.6%).

## Discussion

### Principal findings

Appreciable differences exist in the prevalence of the selected health conditions in the two data sources analysed, in which information was either self-reported (HS) or recorded by a medical practitioner (EHR). There are sex-based differences, with a higher prevalence of the selected health conditions in women. Age-related differences were identified in the prevalence of multimorbidity. Among the elderly, the prevalence is similar in both data sources. In younger patients, however, the multimorbidity prevalence is significantly higher in the HS data than in the EHR. Independent of the method used to measure morbidity, multimorbidity is widely prevalent and may affect at least 22% of younger patients (ages 15–44). Especially in these younger patients, self-report appears to be more sensitive to identifying symptoms-based conditions. The subgroup of the population who are selected for the periodic survey and provide self-reports on the selected health conditions may not visit their primary care services frequently, or for other reasons these conditions may not be recorded as often in the EHR database.

Musculoskeletal health problems (neck and back pain, rheumatism diseases) and other health problems (varicose veins, migraine or frequent headaches, haemorrhoids and allergies) were more frequently identified in the HS. Although it is not clear why these problems may be under-recorded in the EHR, it is likely that health professionals more consistently register those health problems that require continuous treatment, testing and referral to specialized care. It is possible that these diseases are not always judged to be clinically relevant [21].

Our data suggest that conditions requiring diagnostic tests are not over-represented in the EHR compared to HS data. In sharp contrast, three of four symptoms-based health problems have a higher prevalence in the HS.

### Comparison with other studies

Prevalence of health problems as obtained from the HS data is consistent with results from another study of HS data [22]. Our estimate of the prevalence of health problems registered in the EHR is also consistent with those obtained in other population-based studies in Spain [22-25].

Multimorbidity increased with age, especially in older people (at least 83% in those aged 65 or older), with rates similar to published data that include these age groups [26]. The high number of health problems (average of 3.6) perceived in this age group should be noted.

Our hypothesis that conditions based on test results will be more prevalent in EHR than in HS data was confirmed for cardiac disease, diabetes mellitus, and malignant tumours; two conditions, hypertension and myocardial infarction, had similar estimated prevalence in both sources. For the remaining 22 selected health conditions the hypothesis was not confirmed. Our second hypothesis, that symptomatic conditions would be more frequently recorded in the HS than in EHR data, was confirmed, except in the case of mental disorders, prostatic disorders and skin diseases. There are several possible explanations for these results. First, less severe conditions may not be recorded in the EHR and individuals may overstate their condition in the HS. Among the problems discussed during one medical consultation, only those requiring a prescription or a specific action tend to be codified [27]. Therefore, the HS may detect less complex problems. Health conditions more frequently registered in EHR could be conditioned by their severity (cardiac disease and malignant tumour) or by the fact that some chronic conditions are part of the primary care objectives established by the institution (diabetes mellitus and hypertension). Of the three conditions that do not follow the second hypothesis (mental disorders, prostatic disorders and skin diseases), a possible explanation is that these conditions carry more stigma than others and therefore are not as readily reported to an interviewer.

We found a few studies in the international literature that compare self-reports and health records for multiple diseases [4,10]; the most symptomatic conditions were more reflected in HS in approximately half of the chronic conditions in a Spanish article [10]. An Italian study compared four chronic conditions and obtained similarities between two sources in diabetes and hypertension and discrepancies in COPD and gastroduodenal ulcer, concluding that those conditions with more clear diagnostic criteria showed more relevant similarities between the two data sources [28].

Other studies, each focussing on specific health problems, identified good agreement between data sources for malignant tumours [28], diabetes and hypertension, but not for rheumatologic problems [29], prostatic disorders [30] and skin diseases [31]. Our research is the first to compare multimorbidity in self-reported and EHR data on a wide range of diagnoses and based on a large clinical database.

Problems in the mental sphere in the youngest age group (<44 years), the emergence of hypertension, diabetes and hyperlipidaemias in middle age and the onset of prostatic pathology in men and osteoarticular in women older than 65 synthesized the distribution of conditions throughout the lifespan. Hypertension was commonly combined with other conditions, as in other studies [32]. Overall, the cardiovascular diseases (with hypertension in the lead), musculoskeletal disorders, mental disorders and metabolic problems were the most prevalent. One difference from other studies is the cluster of mental diseases (depression/anxiety and mental disorders) as the sixth most common pair of health problems. These two categories of mental disorders constituted more than one sixth of the estimated total prevalence of morbidity, surpassed only by the combinations of cardiovascular and metabolic disorders. These differences could be explained because some studies excluded mental disease [33,34] or grouped psychiatric problems differently. Similarly, we did not include obesity, which was analysed in other studies.

### Strengths and limitations

The main limitation is that we could not link responses in the HS with corresponding individual EHR data. Therefore, we were comparing estimates from two different samples, with different data collection methods. The confidence intervals are adjusted by the multistage sampling in the HS but not the EHR data, in which the individual patient is the unit of analysis. Moreover, we can’t estimate how much variability can be attributed to each source of variation (sampling frame and data collection). The subgroup of the population selected for the periodic survey and who provide self-reports on the selected health conditions may not visit their primary care services frequently, or for other reasons these conditions may not be recorded as often in the EHR database. The EHR sample consisted of individual patient data, recorded by GPs who meet established quality standards for coding and research-ready data. These health professionals were specifically selected for their record of quality in coding the selected diseases [17].

However, we established that both the HS and EHR data sets were broadly comparable with the general population, and that there is a similar distribution by sex and age group in both samples.

We analysed only the health problems included in the HS. This renders comparison difficult with other studies focusing on different sets of conditions [26,35]. A recent review found 39 different indexes to measure multimorbidity, with an average of 18.5 health problems included [36]. We analysed 27 health problems, more than the 12 frequent diagnoses of chronic diseases that have been suggested to be ideal for the study of multimorbidity [11].

The HS data was based on self-perceived health status, while EHR registered only the health professional’s final diagnosis, codified following ICD-10 classification. The mapping process involved the clinical consensus of four experienced primary care physicians, who identified all ICD 10 codes relevant to each condition included in the HS. Therefore, an effort to define the origin of the differences between the two sources of data is influenced by various factors. There are many factors affecting both self-perceived and officially recorded health problems [35]. A positive association has been established between self-reported health and the use of health care services, especially in older people [37]. Nevertheless, self-reported questionnaires are based on the ability to recall past events [38] and there are substantial discrepancies between self-reported and administrative data, especially among older adults [39]. It is also known that several determinants can condition how a population defines their own health, such as educational level [40].

Finally, the use of existing databases has some inherent disadvantages, such as possible data quality issues and the difficulty of processing potential confounders [41]. This is the reason behind our restrictive quality criteria for the inclusion of medical records [14,17]. There is no indication that these eventualities affected our results.

### Implications for clinical and policymakers

Health surveys provide information on health status that is not reflected in medical records. One explanation is that patients themselves may consider that some health disorders are not important enough to use health services, but when they are specifically asked to report them the probability of expressing these problems improves. The highest differences in prevalence of conditions are gender-related and could be explained because men use health services less than women [42], although recent studies examining consultations for common symptoms by sex are in line to dismantle this paradigm [43].

On the other hand, a set of papers compared methods of measurement that are self-report versus administrative data [44,45] or medical records [46] with regard to outcomes, and concluded that self-reporting increases the predictive accuracy.

Incorporating self-information in multimorbidity studies allows patients to provide their perception of those problems that interfere more in their everyday lives and are in line with the concept of the Evidence-Based Patient [47].

### Future research

Since we have found several disparities between registered and self-reported health data, future research on multimorbidity should not be based only on information from medical records but must take into account the patient perspective. The challenge in future research will be the incorporation of perceived diseases in databases, so that the diagnosis “below the iceberg” can be minimized. This approach is necessary to defining the concept of multimorbidity among researchers and health professionals, in order to propose an homogeneous index of multimorbidity to be applied in clinical practice, in clinical research and in epidemiology and health management.

## Conclusions

Prevalence of multimorbidity differs depending on whether the information is obtained from self-reported health status or a medical record. There are sex-based differences, with a higher prevalence of the selected health conditions in women. Regardless of the method used to measure morbidity, multimorbidity is widely prevalent and may affect at least 22% of the youngest patients (ages 15–44). Age-related differences in multimorbidity prevalence were identified, especially in this youngest age group. The prevalence of self-reported multimorbidity was significantly higher in HS data among these patients. The difference attenuates with age, and prevalence was similar in both data sources for elderly patients.

Health surveys detect musculoskeletal problems more frequently, as well as other conditions that might be considered minor. In general, symptoms-based chronic conditions are more reflected in HS than in EHR data. The HS and EHR data provide substantially different estimates of multimorbidity, and this should be taken into account for the design of future studies.

## Abbreviations

EHR: Electronic health records; HS: Health survey; PCP: Primary care practices; GP: General practitioner; ICD-10: International Statistical Classification of Diseases; IDIAP: Institut Universitari d’Investigació en Atenció Primària Jordi Gol (IDIAP Jordi Gol). (Primary Health Care University Research Institute Jordi Gol); COPD: Chronic obstructive pulmonary disease.

## Competing interests

This work has been funded by the Ministry of Science and Innovation through the Instituto Carlos III as part of the Preventive Services and Health Promotion Network (redIAPP), by ISCiii-RETICS (RD06/0018), by internal research grants, and by a 2011–2012 scholarship that aims to promote research in Primary Health Care by health professionals who have completed their specialty training, awarded by Institut Universitari d’Investigació en Atenció Primària Jordi Gol (IDIAP Jordi Gol). The funders had no role in the study design, collection, analysis and interpretation of data, writing of the manuscript and decision to submit for publication.

## Authors’ contributions

CV, EH, JMV, BB, MF, PB, and MM drew up the study protocol and structured the bibliographical search. EH, MF, and PB carried out data collection. CV, QF, EH, MM and JMV conducted the analysis and interpretation of the initial results. All authors contributed ideas, interpreted the findings and reviewed rough drafts of the manuscript. All authors approved the final versions of all manuscripts. CV is the head of the Catalan study.

## Pre-publication history

The pre-publication history for this paper can be accessed here:

http://www.biomedcentral.com/1471-2458/13/251/prepub

## Supplementary Material

Additional file 1: Appendix 1 International Classification of Diseases (ICD-10) Codes Assigned to the 27 Health Conditions Reported in the Health Survey of Catalonia. Click here for file

Additional file 2: Appendix 2 Prevalence of 27 Selected Conditions in the Electronic Health Records by Sex and Age Group. Click here for file
