# Supplementary material for: Comparison of the information provided by electronic health records data and a population health survey to estimate prevalence of selected health conditions and multimorbidity
Source: BMC Public Health. 2013 Mar 21;13:251. doi: 10.1186/1471-2458-13-251 (PMC3659017; doi:10.1186/1471-2458-13-251)
Supplement: Additional file 1: Appendix 1 — International Classification of Diseases (ICD-10) Codes Assigned to the 27 Health Conditions Reported in the Health Survey of Catalonia. [file 1471-2458-13-251-S1.docx]

**Appendix 1. International Classification of Diseases (ICD-10) Codes Assigned to the 27 Health Conditions Reported in the Health Survey of Catalonia**

*Except myocardial infarction. † Chronic obstructive pulmonary disease. ‡ Except depression and anxiety. § Excluding varicose veins.
