# Supplementary material for: Comparison of the information provided by electronic health records data and a population health survey to estimate prevalence of selected health conditions and multimorbidity
Source: BMC Public Health. 2013 Mar 21;13:251. doi: 10.1186/1471-2458-13-251 (PMC3659017; doi:10.1186/1471-2458-13-251)
Supplement: Additional file 2: Appendix 2 — Prevalence of 27 Selected Conditions in the Electronic Health Records by Sex and Age Group. [file 1471-2458-13-251-S2.docx]

**Appendix 2. Prevalence of 27 Selected Conditions in the Electronic Health Records by Sex and Age Group**

|  | **ALL** | | | | | **MALE** | | | | | **FEMALE** | | | | |
| --- | --- | --- | --- | --- | --- | --- | --- | --- | --- | --- | --- | --- | --- | --- | --- |
|  | **15-44** | **45-64** | **65-74** | **≥75** | **TOTAL** | **15-44** | **45-64** | **65-74** | **≥75** | **TOTAL** | **15-44** | **45-64** | **65-74** | **≥75** | **TOTAL** |
| **Anaemia** | 3.2 | 4.7 | 4.9 | 11.2 | *4.6* | 0.5 | 1.4 | 4.3 | 10.7 | *2.0* | 5.9 | 7.6 | 5.5 | 11.5 | *7.0* |
| **Asthma** | 4.4 | 3.3 | 4.4 | 4.5 | *4.1* | 4.3 | 2.2 | 2.6 | 2.3 | *3.4* | 4.5 | 4.3 | 6.0 | 5.9 | *4.8* |
| **Back pain** | 10.4 | 17.2 | 17.8 | 15.0 | *13.6* | 9.6 | 15.2 | 15.8 | 13.9 | *12.2* | 11.2 | 19.1 | 19.6 | 15.6 | *14.9* |
| **Cardiac disease*** | 1.0 | 5.1 | 15.8 | 29.8 | *6.6* | 1.2 | 6.3 | 18.3 | 32.4 | *8.1* | 0.8 | 4.0 | 13.6 | 28.2 | *6.3* |
| **Cataracts** | 0.1 | 1.3 | 8.8 | 19.1 | *3.2* | 0.1 | 1.3 | 8.1 | 17.6 | *2.7* | 0.1 | 1.3 | 9.3 | 20.2 | *3.7* |
| **Cerebrovascular disease** | 0.1 | 0.9 | 3.3 | 6.7 | *1.3* | 0.1 | 1.3 | 4.5 | 8.4 | *1.6* | 0.1 | 0.6 | 2.3 | 5.6 | *1.1* |
| **Chronic allergies** | 3.7 | 2.9 | 2.5 | 1.8 | *3.2* | 3.6 | 2.3 | 2.3 | 1.9 | *3.0* | 3.9 | 3.4 | 2.7 | 1.7 | *3.4* |
| **Chronic constipation** | 1.6 | 2.3 | 4.3 | 8.1 | *2.7* | 0.8 | 1.2 | 3.2 | 7.2 | *1.7* | 2.4 | 3.4 | 5.2 | 8.6 | *3.7* |
| **COPD†** | 0.8 | 3.7 | 9.8 | 13.0 | *3.8* | 0.8 | 5.2 | 15.8 | 22.7 | *5.4* | 0.8 | 2.3 | 4.6 | 6.6 | *2.3* |
| **Depression or anxiety** | 12.3 | 20.2 | 19.0 | 18.1 | *15.9* | 8.5 | 12.2 | 10.3 | 9.9 | *9.9* | 16.1 | 27.5 | 26.5 | 23.4 | *21.3* |
| **Diabetes mellitus** | 0.9 | 9.0 | 21.3 | 23.9 | *7.6* | 1.1 | 11.7 | 24.4 | 26.1 | *8.6* | 0.7 | 6.6 | 18.6 | 22.5 | *6.8* |
| **Haemorrhoids** | 2.1 | 4.3 | 5.4 | 4.6 | *3.3* | 2.1 | 4.1 | 4.9 | 4.6 | *3.2* | 2.2 | 4.5 | 5.8 | 4.7 | *3.5* |
| **Hypercholesterolemia** | 2.6 | 15.0 | 22.3 | 18.7 | *9.8* | 2.9 | 14.8 | 20.3 | 16.4 | *9.2* | 2.2 | 15.2 | 24.0 | 20.3 | *10.3* |
| **Hypertension** | 2.1 | 24.6 | 54.5 | 66.8 | *20.4* | 2.7 | 26.9 | 53.2 | 61.7 | *19.6* | 1.5 | 22.5 | 55.6 | 70.1 | *21.2* |
| **Mental disorders**‡ | 12.9 | 18.1 | 14.9 | 15.3 | *14.8* | 13.8 | 19.8 | 16.9 | 14.9 | *15.9* | 12.1 | 16.4 | 13.1 | 15.6 | *13.8* |
| **Migraine or frequent headaches** | 5.8 | 5.5 | 3.8 | 2.5 | *5.2* | 3.8 | 2.7 | 2.0 | 1.6 | *3.1* | 7.9 | 8.0 | 5.3 | 3.1 | *7.1* |
| **Myocardial infarction** | 0.1 | 2.0 | 5.9 | 9.3 | *2.2* | 0.1 | 3.5 | 9.6 | 13.8 | *3.2* | 0.0 | 0.6 | 2.8 | 6.3 | *1.2* |
| **Neck pain** | 4.9 | 7.2 | 6.6 | 5.0 | *5.7* | 3.3 | 4.5 | 5.0 | 4.4 | *3.9* | 6.4 | 9.7 | 8.1 | 5.4 | *7.4* |
| **Malignant tumours** | 0.6 | 3.9 | 9.3 | 12.5 | *3.6* | 0.5 | 3.3 | 10.9 | 17.4 | *3.7* | 0.7 | 4.4 | 7.8 | 9.3 | *3.5* |
| **Osteoarthritis, arthritis or rheumatism** | 1.2 | 12.5 | 30.8 | 39.0 | *11.3* | 1.2 | 9.9 | 24.1 | 31.4 | *8.5* | 1.2 | 15.0 | 36.6 | 43.9 | *13.8* |
| **Osteoporosis** | 0.1 | 4.2 | 12.2 | 13.8 | *3.9* | 0.1 | 0.5 | 1.7 | 2.9 | *0.6* | 0.1 | 7.6 | 21.3 | 20.8 | *6.9* |
| **Circulation disorders** ¶ | 0.2 | 1.0 | 2.6 | 4.0 | *1.1* | 0.1 | 1.4 | 4.1 | 6.3 | *1.4* | 0.3 | 0.6 | 1.4 | 2.5 | *0.7* |
| **Peptic ulcers** | 0.4 | 2.4 | 3.9 | 4.0 | *1.7* | 0.6 | 3.3 | 5.8 | 6.0 | *2.3* | 0.3 | 1.6 | 2.3 | 2.6 | *1.1* |
| **Prostatic disorders** | NA | NA | NA | NA | *NA* | 0.2 | 7.6 | 31.1 | 43.9 | *9,1* | NA | NA | NA | NA | *NA* |
| **Skin diseases** | 9.8 | 8.7 | 10.9 | 11.0 | *9.7* | 8.6 | 8.1 | 11.5 | 12.4 | *9.1* | 11.0 | 9.4 | 10.5 | 10.0 | *10.4* |
| **Thyroidal diseases** | 1.5 | 3.5 | 4.2 | 4.2 | *2.6* | 0.5 | 0.9 | 1.5 | 1.9 | *0.8* | 2.5 | 5.8 | 6.4 | 5.7 | *4.2* |
| **Varicose veins** | 2.0 | 6.9 | 11.7 | 13.8 | *5.6* | 0.8 | 2.9 | 4.9 | 6.6 | *2.3* | 3.1 | 10.6 | 17.6 | 18.4 | *8.6* |

NA: Not Applicable. *Excluding myocardial infarction. † Chronic obstructive pulmonary disease. ‡ Except depression and anxiety. § Excluding varicose veins.
